# Case report: Emergency treatment of late-presenting congenital diaphragmatic hernia with tension gastrothorax in three Chinese children

**DOI:** 10.3389/fped.2023.1115101

**Published:** 2023-02-01

**Authors:** Rui Guo, Lina Zhang, Shisong Zhang, Hongxiu Xu, Yunpeng Zhai, Huashan Zhao, Longfei Lv

**Affiliations:** ^1^ Department of Thoracic and Tumor Surgery, Children’s Hospital Affiliated to Shandong University, Jinan, China; ^2^ Department of Thoracic and Tumor Surgery, Jinan Children’s Hospital, Jinan, China; ^3^ Department of Emergency, Zhangqiu Dist Peoples Hospital, Jinan, China

**Keywords:** congenital diaphragmatic hernia, tension gastrothorax, emergency treatment, latepresenting, endoscopic surgery

## Abstract

**Background:**

Congenital diaphragmatic hernia (CDH) is a scarce birth defect. It is called late-presenting CDH when symptoms are found after 1 month of life. The clinical manifestations of late-presenting CDH are diverse, among which the most fatal is the cardiac arrest caused by tension gastrothorax. The disease is rare, can easily lead to death owing to improper emergency treatment. This report illustrates the emergency treatment of late-presenting CDH with tension gastrothorax in three Chinese children.

**Case reports and management:**

Three children presented to emergency room with a sudden dyspnea, diagnosed accurately by x-ray or computed tomography. In case 1, the gastric tube could not be inserted at the first attempt, and the child cried incessantly. Cardiac arrest occurred when the gastric tube was re-inserted. After cardiopulmonary resuscitation and placement of a thoracic drainage tube, a large amount of gas and stomach contents were drained. Laparoscopic surgery was performed. The patient died of sepsis. In case 2, the gastric tube could not be inserted at the first attempt; consequently, emergency surgery was considered instead of retrying. After the patient was anesthetized, a gastric tube was successfully placed. Subsequently, a large amount of gas and gastric contents was drained, and thoracoscopic surgery was performed. The patient recovered evenly. In case 3, the gastric tube was successfully inserted at the first attempt; however, the vital signs were unstable due to poor drainage of the gastric tube. We injected 20 ml of iohexol into the stomach tube for angiography and dynamic chest film monitoring. After adjusting the position of the stomach tube, the stomach collapsed completely. Thoracoscopic surgery was performed. The patient recovered evenly.

**Conclusion:**

Early diagnosis is essential for children with late-presenting CDH complicated by tension gastrothorax. Fully collapsing the stomach is a key step in emergency treatment. In addition, gastric tube insertion is the first choice. In children with difficulty in gastric tube placement at the first attempt, the gastric tube can be placed under anesthesia, and emergency surgery performed simultaneously. Endoscopic surgery can be the first choice in cases of complete stomach collapse.

## Background

Congenital diaphragmatic hernia (CDH) is a developmental closure defect, resulting in discontinuity of the diaphragm. This allows abdominal viscera to herniate into the chest (1). Many abdominal organs pour into the chest, leading to pulmonary dysplasia and respiratory distress after birth, requiring comprehensive multidisciplinary treatment (2). Although some children suffer from CDH, there are no abnormalities on B-ultrasound during pregnancy and no symptoms in the neonatal period. Those with symptoms after 1 month of life are called late-presenting CDH, accounting for approximately 10% of CDH cases (3). The clinical manifestations of late-presenting CDH are diverse, among which the most fatal is the cardiac arrest caused by tension gastrothorax. The disease is rare, has an acute onset, and can easily lead to death owing to improper emergency treatment. According to a PubMed literature search, there are few reports of late-presenting CDH with tension gastrothorax, and there are no reports of Chinese children in the English literature (4, 5). This report illustrates the emergency treatment of late-presenting CDH with tension gastrothorax in three Chinese children.

## Case description

### Case 1

**Figure 1:**
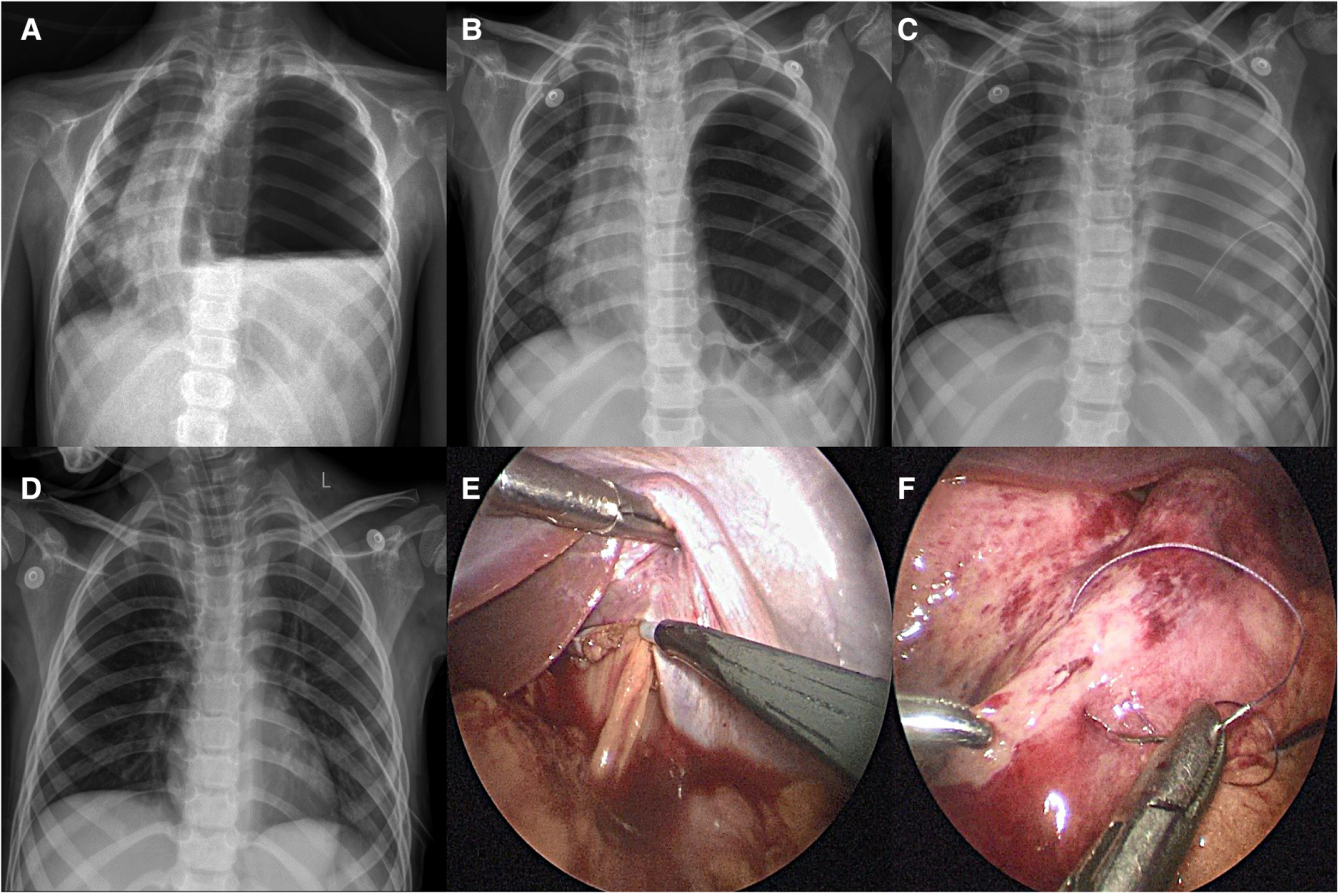
Case 1: preoperative and postoperative x-ray findings and intraoperative conditions: (**A**) encapsulated gas accumulation with mediastinal displacement; (**B**) encapsulated gas accumulation still exists after re-placing the thoracic drainage tube; (**C**) after adjusting the thoracic drainage tube, the wrapped gas accumulation disappeared, but the left costophrenic angle was vague, indicating the presence of pleural effusion; (**D**) after the operation, the diaphragm recovered to normal position and the lung expanded well; (**E**) hemorrhagic effusion in the abdominal cavity, obvious gastric edema and expansion, and enlarged diaphragm incision; (**F**) repair iatrogenic gastric perforation.

A male child, aged 4 years and 5 months, suffered a sudden dyspnea for 8 h and went to emergency room. Perinatal and neonatal examinations were normal. The child had been in good health since birth. A general examination revealed nasal flaring, positive triple concave sign, tachycardia (145 beats/min) and tachypnea (55 breaths/min). His oxygen saturation level was 85% in ambient air. Breath sounds were absent from the left side on auscultation. The x-ray showed an air-fluid level, suggesting an encysted hydropneumothorax with a mediastinal shift and a diagnosis of tension gastrothorax (Figure 1A). We attempted to decompress the stomach by inserting a gastric tube but failed. The child cried incessantly and experienced cardiac arrest when the gastric tube was re-placed. Cardiopulmonary resuscitation was performed, and a thoracic drainage tube was placed to drain a large amount of gas and stomach content. Tracheal intubation was performed to support respiration. The heart rate fluctuated between 110‒130 times/min. The x-ray showed that there was still wrapped-gas accumulation, which disappeared after adjusting the position of the closed thoracic drainage tube. However, the left costophrenic angle was unclear, indicating pleural effusion (Figure 1B,C). Emergency laparoscopy revealed a left posterolateral diaphragmatic defect, through which the stomach, spleen, and colon herniated into the left thorax. Gastric edema and dilatation were evident. After enlarging the diaphragm incision, all organs were brought into the abdominal cavity; iatrogenic gastric perforation and diaphragmatic defects were repaired (Figure 1E,F). Postoperative x-ray was normal (Figure 1D). However, the patient suffered from a high fever postoperatively, developed septic shock, and died of sepsis.

### Case 2

**Figure 2:**
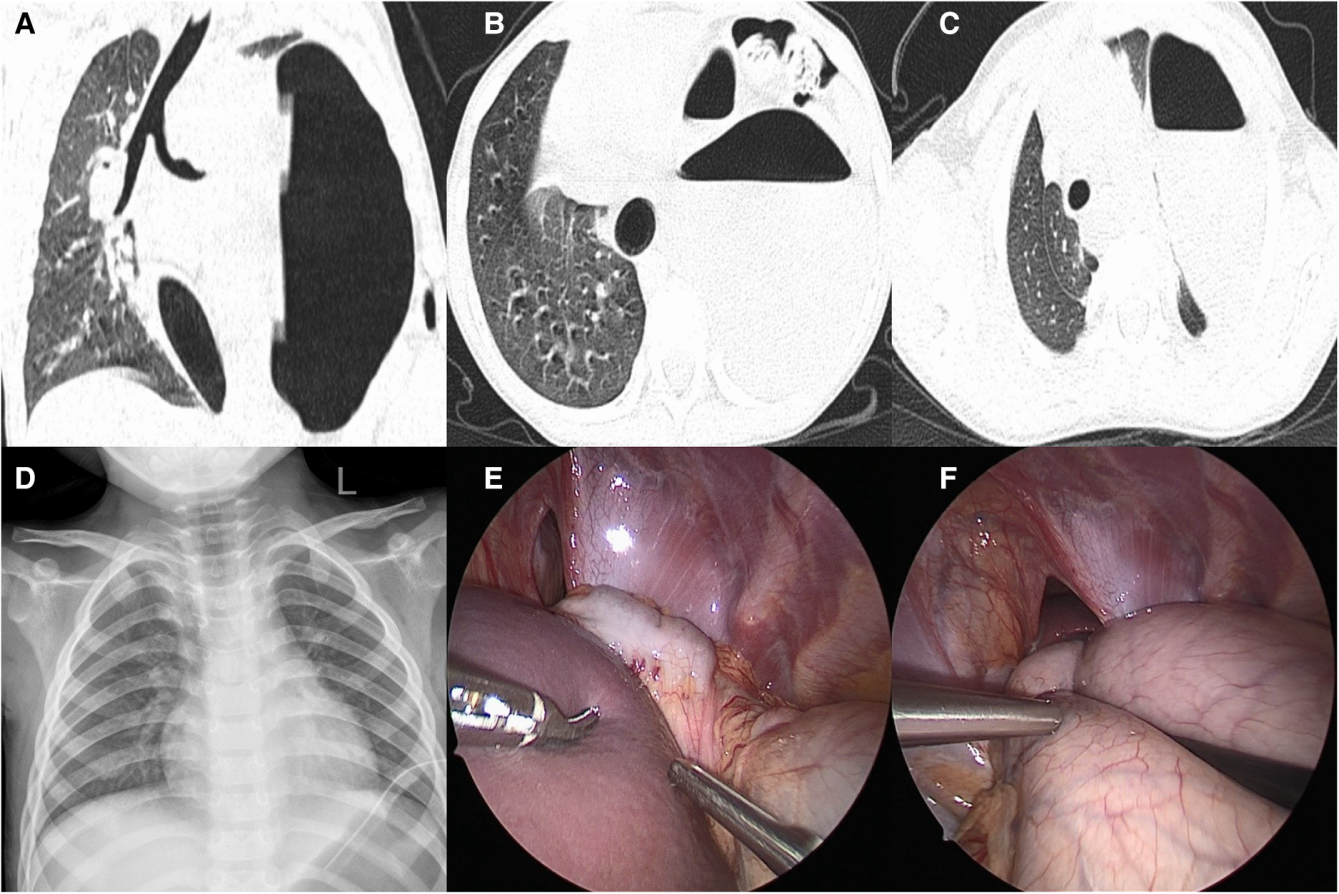
Case 2: preoperative CT, postoperative x-ray manifestations, and intraoperative conditions: (**A**) encapsulated gas accumulation with mediastinal displacement; (**B**) stomach and part of colon herniated into the thoracic cavity; (**C**) left lung was atelectasis and compressed to the upper of the stomach; (**D**) after the operation, the diaphragm recovered to normal position and the lung expanded well; (**E**) the hernia sac mouth is small, and the spleen and part of colon herniated into it; (**F**) the spleen had returned to the abdominal cavity and presented the hollow and herniated stomach in the thoracic cavity.

A male child, aged 6 years, suffered a sudden dyspnea for 8 h and went to emergency room. Perinatal and neonatal examinations were normal. The child went to a doctor for “bronchitis” 1 year ago, and no abnormality was found on auscultation. General examination showed nasal flaring, positive triple concave sign, tachycardia (140 beats/min) and tachypnea (53 breaths/min). His oxygen saturation was 87% in ambient air. x-ray and computed tomography (CT) showed that the stomach herniated into the thoracic cavity, gastric dilatation resulted in mediastinal deviation, and the left lung was atelectatic and located in the upper part of the stomach. The diagnosis was tension gastrothorax (Figure 2A‒C). We chose gastric tube implantation initially; however, this was unsuccessful. Because the patient cried incessantly, we did not re-attempt gastric tube insertion. Subsequently, gastric tube insertion was performed after anesthesia when the child was in a resting state. Thoracoscopic surgery is feasible if gastric tube insertion is successful and the stomach decompresses completely. The herniated organ can be returned to the abdominal cavity, and the diaphragmatic hernia is repaired; if the gastric tube insertion fails, a thoracotomy should be performed immediately. We opened the gastric wall under direct vision, stretched the aspirator into the gastric cavity to absorb gas and gastric contents for decompression, repaired the gastric wall, returned the herniated organ to the abdominal cavity, and repaired the diaphragmatic hernia. In this case, after anesthetization, we successfully placed a gastric tube, leading to the production of a large amount of gas and gastric contents of approximately 400 ml. Subsequently, thoracoscopic surgery was performed. We found that the colon, spleen, and stomach herniated from a small hernial orifice during the surgery. All herniated organs were brought into the abdominal cavity, and the diaphragmatic defects were repaired (Figure 2E,F). The postoperative radiographs were normal (Figure 2D). The patient recovered evenly and was followed up for a year postoperatively; there was no report of vomiting, abdominal pain, or respiratory distress.

### Case 3

**Figure 3:**
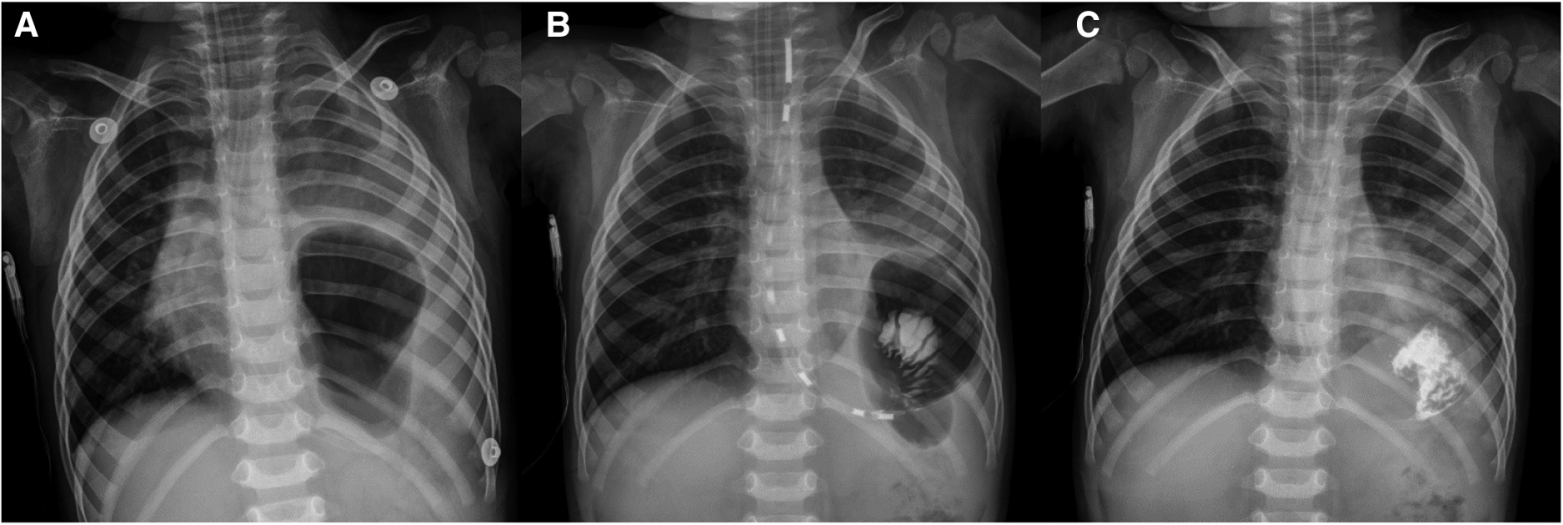
Case 3: x-ray manifestations before and after operation: (**A**) encapsulated gas accumulation with mediastinal displacement; (**B**) the gastric tube is not bent, and its distal end is located in the stomach; (**C**) after adjusting the gastric tube for the complete decompression, there was no encapsulated gas accumulation with mediastinal displacement.

A male child, aged 2 years and 4 months, suffered a sudden dyspnea for 3 h and went to emergency room. Perinatal and neonatal examinations were normal. The child has been in good health since birth. A general examination revealed nasal flaring, positive triple concave sign, tachycardia (162 beats/min) and tachypnea (61 breaths/min). His oxygen saturation level was 75% in ambient air. The patient experienced sudden cardiac arrest. Cardiopulmonary resuscitation was immediately performed; endotracheal intubation was performed to support respiration. After inserting the gastric tube, a large amount of gas and gastric contents (approximately 200 ml) was discharged. Electrocardiogram (ECG) monitoring showed a heart rate of 145 beats/min and percutaneous oxygen saturation of 92%. The child was immediately transferred to the intensive care unit for further treatment. Invasive arterial pressure monitoring showed a blood pressure of 65/42 mmHg, indicating low blood pressure and a small pulse pressure difference. The x-ray showed an air-fluid level, suggesting an encysted hydropneumothorax with a mediastinal shift and a diagnosis of tension gastrothorax (Figure 3A). Because the drainage of the gastric tube was not smooth, and the decompression effect was not satisfactory, it was necessary to check whether the position of the gastric tube was appropriate and adjust it. After injecting 20 ml of iohexol into the stomach tube, we immediately performed an x-ray and found that the stomach tube was not bent, and the distal end was located in the stomach (Figure 3B). We considered the drainage not smooth because the stomach tube was attached to the stomach wall. After pulling out the 5 cm-stomach tube, a large amount of gas and a stomach content of approximately 150 ml were smoothly drained. The x-ray showed no encapsulated hydropneumothorax and a medial shift (Figure 3C). Blood pressure increased to 99/48 mmHg, and percutaneous oxygen saturation was 100%. Because the stomach had fully collapsed, we chose thoracoscopic surgery to return all herniated organs into the abdominal cavity and repair diaphragmatic defects. The postoperative radiographs were normal. The patient recovered evenly and was followed up for 3 months postoperatively; there were no reports of respiratory distress.

## Discussion

The incidence of CDH varies from 0.8 to 5 in every 10,000 births (6). When the diaphragmatic hernia is small, it is protected by the liver on the right and the spleen on the left; therefore, there are usual no symptoms in the recent months to years after birth; patients usual have normal chest radiographs, which are called late-presenting CDH (7, 8). The occurrence of late-presenting CDH is rare and presents with various symptoms, such as cough, dyspnea, chest pain, abdominal pain, and vomiting. In severe cases, patients may suffer serious respiratory or cardiopulmonary failure. This makes the diagnosis of late-presenting CDH challenging: it is often mistaken for other conditions (7–10). Among them, tension gastrothorax complicated by late-presenting CDH is a potentially life-threatening condition and must be diagnosed immediately.

The pathology of tension gastrothorax was first described in 1984 (11). Five steps are necessary to develop a tension gastrothorax: (1) the existence of a diaphragmatic defect, (2) increased intra-abdominal pressure, (3) prolapse of the stomach into the thorax, (4) a functional change in the gastroesophageal junction (by way of an abnormal angulation), and (5) a reduction in cardiac output resulting from mediastinal shift (4, 12). In late-presenting CDHs, it is difficult for the stomach herniating into the thoracic cavity because of the small opening hernia obstructed by the spleen. In rare cases, a sudden increasing abdominal pressure causes herniation of the spleen and stomach into the chest. The hernial stomach is close to the esophagus, and the gastroesophageal junction forms an acute angle, thus acting as a one-way valve. Swallowed secretions and air can enter into the stomach but are difficult to be discharged, which causes the stomach expanding worsening. Children cannot express their illness, and their illness is often ignored. They cry incessantly because of physical discomfort; therefore, an increasing amount of gas is absorbed into the stomach and cannot be discharged, resulting in rapid disease progression. Respiratory distress and cardiac arrest are very likely if not handled in time.

The clinical manifestations of tension gastrothorax and tension pneumothorax are very similar. If the diagnosis is mistaken, the mortality will be greatly increased. Therefore, it is very important to distinguish the above diagnosis by correctly interpreting the chest x-rays. The radiological findings of a tension gastrothorax include a large air-filled structure with or without a fluid level in the left hemithorax, a superior rim formed by a compressed ipsilateral lung and stomach wall, lack of a stomach bubble in the left upper quadrant, poorly-defined left hemidiaphragm, and a mediastinal shift to the right. Although a mediastinal shift to the right is evident in a left-sided tension pneumothorax, the following features will allow a clear-cut distinction from tension gastrothorax: the entire left lung is centrally compressed and surrounded by intrapleural air, the lateral sinus is free, and the left (depressed) hemidiaphragm is well-defined (5, 10). The diagnosis can be further clarified when combined with a CT examination (7).

The full collapse of the stomach is the key to emergency treatment of tension gastrothorax, in which gastric tube placement is the first choice; however, it is challenging. The angle formed at the gastroesophageal junction makes it difficult to place the gastric tube; repeated gastric tube insertion may be fatal in children. Because children do not cooperate with the operation, they constantly cry during gastric tube insertion. Repeated inhalation of gas into the stomach that cannot be discharged will progress and aggravate the disease, leading to cardiac arrest. In addition, serious mediastinal deviations increase the difficulty of cardiopulmonary resuscitation, leading to death. Even if the gastric tube is successfully inserted, it is often easy for it to adhere to the gastric wall, resulting in poor drainage, unsatisfactory decompression, and fluctuating vital signs. Ng et al. reported on 13 children with tension gastrothorax and cardiac arrest; 69.2% (9 cases) died, including 7 cases of cardiac arrest and 2 of sepsis (4). Some experts have suggested that the stomach could be collapsed by needle puncture or by placing a thoracic drainage tube; however, there are many drawbacks. If the needle enters the second intercostal space, it often fails to penetrate the stomach cavity. Reducing the puncture intercostal space is necessary to improve the success rate. Moreover, only gas or thin gastric juice can be extracted, and the decompression effect is limited. Although thoracic drainage can lead to a large amount of gas and stomach contents to achieve rapid decompression, its drawbacks are evident. If the drainage tube is inserted too deeply, it is easy to attach to the gastric wall, resulting in poor drainage; the insufficiently collapsed stomach causes fluctuations in vital signs. If the drainage tube is inserted too shallowly, it can easily escape from the stomach, and a large amount of stomach contents flow into the chest and abdominal cavity, possibly leading to septic shock and sepsis (13). The first patient experienced cardiac arrest when a gastric tube was repeatedly inserted. Although cardiopulmonary resuscitation was successful and surgical treatment was performed, the patient died of sepsis. Therefore, in the follow-up work, we changed the emergency treatment strategy; for children with successful gastric tube placement at the first attempt, we had to dynamically monitor the position of the gastric tube to ensure that the stomach was fully collapsed. In addition, surgical treatment was feasible after the vital signs were stable. In the third patient, the gastric tube was successfully inserted at the first attempt; however, the vital signs were unstable due to poor gastric tube drainage. We injected a small amount of contrast agent and performed dynamic chest x-ray monitoring, adjusted the position of the gastric tube to make the stomach completely collapse, and performed surgical treatment after stabilizing the vital signs. The postoperative recovery was excellent. Henceforth, we will not repeatedly try to insert the gastric tube for children in whom the first attempt was unsuccessful but perform emergency surgery instead. The child went into a resting state after anesthesia; we re-attempted gastric tube insertion. Endoscopic surgery can be performed if gastric tube insertion is successful and the stomach fully collapses. If implantation fails, a thoracotomy should be performed immediately. The gastric wall should be opened under direct vision, and the aspirator should be stretched into the gastric cavity to absorb gas and gastric contents for decompression. Subsequently, the gastric wall is repaired, the herniated organ is brought into the abdominal cavity, and the diaphragmatic hernia is repaired. In the second patient, we chose emergency surgery after the first gastric tube insertion failed. Although the stomach fully collapsed after the gastric tube was successfully inserted in a resting state, we successfully performed thoracoscopic surgery and achieved an excellent therapeutic effect.

### Conclusion

Late-presenting CDH combined with tension gastrothorax is extremely rare but can easily cause death due to acute disease and improper emergency treatment. However, early diagnosis can improve the treatment success rate. Currently, x-rays and CT can be used to determine a diagnosis quickly. The full collapse of the stomach is a key step in emergency treatment. Gastric tube placement is the first treatment choice. Thoracentesis or thoracic drainage can also be performed during emergencies. Emergency surgery can be performed for children in whom the first attempt to insert a gastric tube failed. After anesthesia, the gastric tube can be placed with the patient in a resting state to minimize the risk of cardiac arrest and reduce mortality. Endoscopic surgery can be the first choice considering the stomach is fully collapsed and vital signs are stable.

## Data Availability

The original contributions presented in the study are included in the article/Supplementary Material, further inquiries can be directed to the corresponding author.
